# Plasma fibrin D-dimer levels correlate with tumour volume, progression rate and survival in patients with metastatic breast cancer

**DOI:** 10.1038/sj.bjc.6600069

**Published:** 2002-02-01

**Authors:** L Y Dirix, R Salgado, R Weytjens, C Colpaert, I Benoy, P Huget, P van Dam, A Prové, J Lemmens, P Vermeulen

**Affiliations:** Oncology Center, AZ St-Augustinus Oosterveldlaan 24, 2610 Wilrijk, Belgium; Department of Pathology, University Hospital Antwerp, Wilrijkstraat 10, 2520 Edegem, Belgium

**Keywords:** coagulation, angiogenesis, metastasis, vascular endothelial growth factor, interleukin-6, fibrinogen

## Abstract

Plasma levels of D-dimer are elevated in cancer patients. Activation of the extrinsic coagulation system and the fibrinolytic cascade within a tumour is thought to be related with growth, invasion and metastasis. We have investigated the relationship between these markers of fibrin metabolism, standard clinicopathological variables and serum levels of angiogenic cytokines in three cohorts: group A (*n*=30) consisted of 30 healthy female volunteers, group B (*n*=23) of consecutive patients with operable breast cancer and group C (*n*=84) of patients with untreated or progressive metastatic breast cancer. Plasma D-dimers, fibrinogen, IL-6, vascular endothelial growth factor and calculated vascular endothelial growth factor load in platelets are clearly increased in patients with breast cancer. D-dimers were increased in nearly 89% of patients with progressive metastatic disease. The level of D-dimers was positively correlated with tumour load (*P*<0.0001), number of metastatic sites (*P*=0.002), progression kinetics (*P*<0.0001) and the cytokines related to angiogenesis: serum vascular endothelial growth factor (*P*=0.0016, Spearman correlation=0.285), calculated vascular endothelial growth factor load in platelets (*P*<0.0001, Spearman correlation=0.37) and serum interleukin-6 (*P*<0.0001, Spearman correlation=0.59). Similarly increased D-dimer levels were positively correlated with increased fibrinogen levels (*P*<0.0001, Spearman correlation=0.38). The association between markers of fibrin degradation in patients with progressive breast cancer suggests that the D-dimer level is a clinically important marker for progression and points towards a relation between haemostasis and tumour progression. A role of interleukin-6, by influencing both angiogenesis and haemostasis, is suggested by these observations.

*British Journal of Cancer* (2002) **86**, 389–395. DOI: 10.1038/sj/bjc/6600069 www.bjcancer.com

© 2002 The Cancer Research Campaign

## 

Tumour growth is considered to be dependent on angiogenesis (Folkman, 1990). Ongoing angiogenesis is recognized by the presence of immature vessels, with endothelial cells displaying a pro-coagulant phenotype (Brock *et al*, 1991; Zucker *et al*, 1998; Benjamin *et al*, 1999). Among the pro-angiogenic cytokines vascular endothelial growth factor (VEGF) has spurred the most interest (Bicknel and Harris, 1991; Carmeliet *et al*, 1996a, Carmeliet and Collen, 1997).

The capacity of VEGF to increase vascular permeability is considered to be critical (van Bruggen *et al*, 1999; Yano *et al*, 2000). Plasma and serum VEGF levels are elevated in a majority of untreated cancer patients and these levels are predictive for the clinically measured tumour doubling kinetics (Dirix *et al*, 1996, 1997). Serum VEGF levels are always much higher than plasma levels (George *et al*, 2000). Different studies have consistently observed a significant correlation between platelet count and serum VEGF of patients with different types of cancer (Möhle *et al*, 1997; Salgado *et al*, 1999; Verheul *et al*, 1999; Vermeulen *et al*, 1999).

Systemic abnormalities of haemostasis in cancer patients have increasingly been recognized, but whether this abnormal haemostasis bears any significance in the pathogenesis of the malignant process remains unclear. Many coagulation factors have been found to be either increased or depressed in plasma of cancer patients and increased markers of fibrinogen degradation are among the most frequently observed haemostatic alterations (Murray, 1991). Several reports have strengthened the concept of a physiological connection between activated coagulation and angiogenesis in human cancer. Previously, the activation of coagulation in cancer was mainly considered to be the result of increased expression of tissue factor (TF), leading to the activation of the extrinsic coagulation pathway (Kakkar *et al*, 1995). Recent data have shown that TF also leads to increased expression of VEGF and decreased transcription of thrombospondin, and inhibitor of angiogenesis (Zhang *et al*, 1994; Contrino *et al*, 1996; Carmeliet *et al*, 1996b). The activation of the coagulation system occurs also in the perivascular region in human cancers and contributes to tumour stroma formation, even at a very early stage of tumorigenesis (Shoij *et al*, 1998; Brown *et al*, 1999).

Similarly, platelet turnover is increased even in the presence of thrombocytosis and an increased platelet number has been shown to be predictive for shorter survival in patients with colorectal and lung cancer (Honn *et al*, 1992; Monreal *et al*, 1998). Others have reported on platelet activation and consumption in patients with solid tumours (Verheul *et al*, 2000).

In an earlier study the serum levels of cytokine IL-6 were found to relate with the total amount of VEGF in serum, and also with the calculated platelet VEGF content (Salgado *et al*, 1999). This is in accordance with the known thrombopoietic and VEGF up-regulating activity of IL-6 (Clarke *et al*, 1996; Cohen *et al*, 1996). IL-6 is considered to have direct angiogenic activity (Motro *et al*, 1990; Mateo *et al*, 1994).

We have attempted to relate levels of D-dimers with the extent of disease and progression rate in patients with breast cancer. We further examined in these patients whether markers of ongoing fibrin degradation showed any relation with markers of angiogenesis.

## MATERIALS AND METHODS

### Patients

Group A is the control group and consists of 30 female volunteers. The 107 female patients with breast cancer are divided into two cohorts; group B includes 23 consecutive patients with newly diagnosed stage I or II disease (Table 1

**Table 1:**
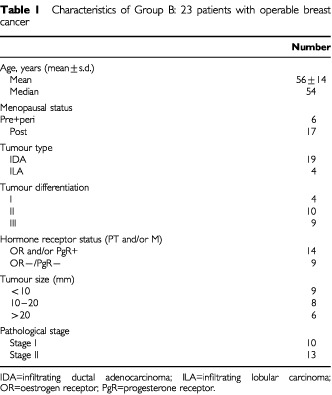
Characteristics of Group B: 23 patients with operable breast cancer

) and group C includes 84 consecutive patients with metastatic disease, either primarily untreated or progressive after therapy (Table 2

**Table 2:**
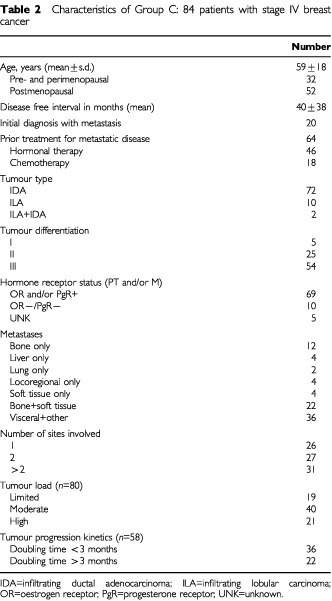
Characteristics of Group C: 84 patients with stage IV breast cancer

). From these 107 (groups B+C) consecutive patients, different clinical and pathological variables were collected; age at diagnosis, current age at sampling time, menopausal state, disease free interval after primary treatment, number and type of prior systemic therapies including adjuvant treatment, histological type, hormone receptor status, number and sites of disease (locally, lymph node and soft tissue, liver, lung, bone, brain and other). Blood samples were collected in group C when untreated metastatic disease was either initially diagnosed or progression of disease was documented radiologically and prior to the initiation of a (new) systemic therapy. For patients in group B sampling occurred at the moment of diagnosis prior to initiation of treatment or biopsy.

### Tumour load

An estimation of the extent of disease was attempted for all group C patients. For bony lesions a whole body technetium scan at the time of data collection was used, with limited disease being equal to 1–2 hot spots, moderate load implying 3–5 separate hot spots and large tumour load more than five separate spots. For liver involvement a standard contrast-enhanced CT-scan and for pulmonary lesions a standard X-ray or a CT-scan were used. These were scored for (a) small volume of disease if <10% of the estimated organ volume was involved with disease, (b) moderate if this was increased up to 10–25%, and (c) large burden was recorded once more than one quarter of either the liver or lung were estimated to be replaced by tumour. Two investigators scored each patient separately. A reliable estimation of tumour load was considered feasible in 80 patients.

### Tumour progression kinetics

Tumour progression was estimated for every patient in group C, if any measurable (in two dimensions) lesion was documented, either clinically or radiologically at the moment of entry into the study and at some point in time during the preceding 3 months. Disease progression was considered to be rapid, when estimated tumour doubling time (duplication of surface area) in any one lesion was less than 3 months. In 58 of the 84 patients an estimation of the kinetics of tumour progression was made. These were divided into a rapid (<3 months) and a slow progressive group (>3 months).

### Blood collection and tests

#### Coagulation tests

Plasma was collected into a 3 ml blood collection tube, containing trisodium (Vacutainer, Becton-Dickinson). Samples were prepared by centrifugation at 2200 **g** for 10 min. Prothrombin time (PT), partial thromboplastin time (PTT) and fibrinogen (FG) were determined on the photo-optical SYSMEX CA6000 Coagulation automate. For the PT determination, a recombinant human tissue factor was used and for the aPTT a phospholipid reagent with particulate activator was applied (micronized silica). Quantitative D-dimer levels were obtained using the enzyme linked fluorescent assay (ELFA) on a mini VIDAS system (bio-Merieux). The assay employs a quantitative sandwich enzyme immunoassay technique combining a bound anti-D-Dimer monoclonal immunoglobulin with an unbound enzyme labelled anti-D-dimer monoclonal immunoglobulin. D-Dimer levels greater than 250 ng ml^−1^ were considered to be elevated. All coagulation parameters are measured on citrated plasma within 2 h of sample collection. Whole blood collection was made by taking a venous blood sample of 3 ml into a blood collecting tube containing EDTA as anticoagulant.

#### Angiogenic cytokines

Simultaneously with the previous blood sampling, another 10 ml of venous blood was drawn into a serum separator tube (Vacutainer, Becton-Dickinson) and allowed to stand for 30 min at room temperature to ensure full clotting. All samples were centrifuged at 3000 **g** for 5 min and the supernatanses were aliquoted and stored at −80°C. Serum levels of bFGF, VEGF and IL-6 were determined using three different enzyme-linked immunosorbent assays (ELISA) (R&D Systems, Minneapolis, MN, USA; Quantikine High Sensitivity human FGF basic; Quantikine human VEGF; Quantikine human IL-6). Within assay reproducibility has been tested before (Dirix *et al*, 1996, 1997; Salgado *et al*, 1999; Vermeulen *et al*, 1999) All samples were run in duplicate. The upper limit of normal for these cytokines was defined by using a 95% confidence limit from the mean value obtained from healthy volunteers. For IL-6 this was 1.3 pg ml^−1^ for VEGF 250 pg ml^−1^ and for bFGF 5.0 pg ml^−1^.

### Ethical committee

This study was approved by the local Ethical Committee and oral informed consent was obtained.

### Statistical analysis

Statistical analysis was performed with the Statview 4.51 software application (Abacus Concepts) on an Apple Macintosh personal computer. Spearman correlation coefficients were used to examine the association between pairs of variables. Comparison of continuous variables in different subgroups were performed by Mann–Whitney *U*-test or by a Kruskal–Wallis test. Relationships between categorical variables were compared using a χ^2^ test. Overall survival was studied by Kaplan–Meier method analysis. Univariate survival curve differences were tested for significance with a log-rank test. A Cox proportional hazards regression model was used to assess the prognostic significance of the parameters taken in association.

## RESULTS

### Patients characteristics

The characteristics of the study population are listed in Tables 1 and 2. A total of 107 blood samples were collected from 107 patients (23 patients of group B, 84 of group C). The 84 patients of group C are part of a total of 140 patients treated during that same period (8 months) with progressive disease. Of those 140 patients, 50 were excluded because progression became apparent within 4 weeks of chemotherapy administration. In the same period two patients were admitted with both progressive disease and a thrombotic event (one arterial thrombosis of the lower limb considered to be a tumour-related event, one patient with deep venous thrombosis and pulmonary embolism). Four patients were excluded because of persisting thrombocytopenia after chemotherapy. Most tumours were infiltrating ductal carcinomas (85.7%). Only 18 patients had been chemotherapy treated for metastatic disease. A majority of patients (46 out of 84, 54%) was progressive under hormonal therapy after either ovarian ablation in 12, tamoxifen in 28, or exemestane in eight. In 20 patients sampling occurred at the time of first diagnosis of metastatic disease, six of these patients were diagnosed to have metastatic disease during their initial staging procedure for breast cancer. The extent of metastasis is shown in detail both with regard to type and number of sites, and including an estimation of tumour load. In the 64 patients with treated metastatic disease, doubling times could be estimated for 58 patients (90%).

### Routine coagulation tests, plasma D-dimer levels and breast cancer stage

Plasma D-dimer levels were increased in the two groups of breast cancer patients (Tables 3

**Table 3:**
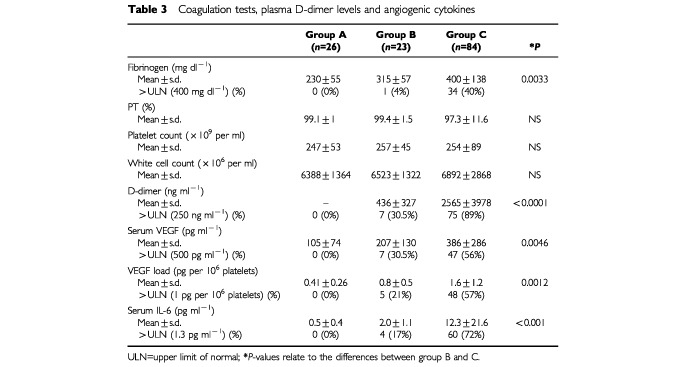
Coagulation tests, plasma D-dimer levels and angiogenic cytokines

and 4

**Table 4:**
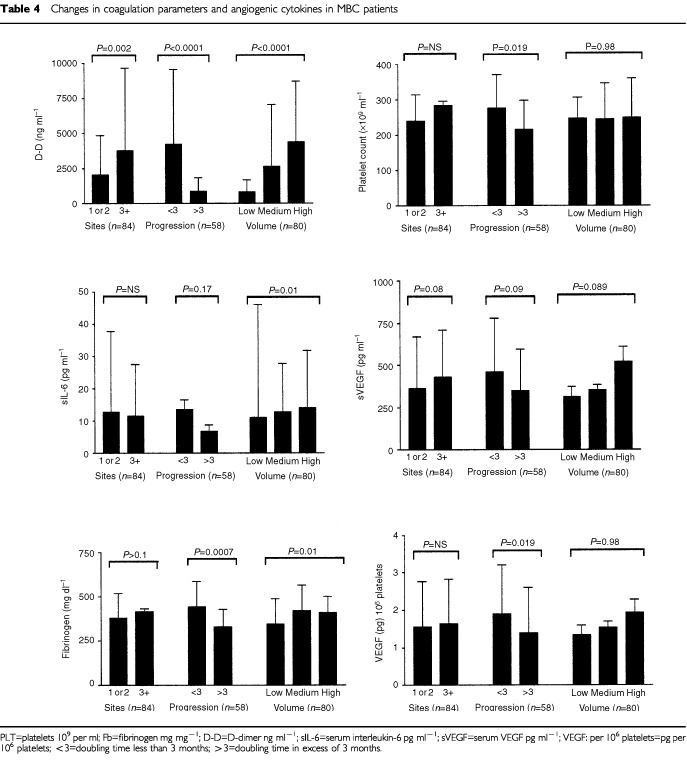
Changes in coagulation parameters and angiogenic cytokines in MBC patients

). The mean D-dimer level for all patients with breast cancer (groups B+C) was 2099±3623 ng ml^−1^ (median 873 ng ml^−1^; range 113–26009; *n*=107). This was significantly higher compared to the volunteer population (*P*<0.0001). Patients with metastatic disease (group C) had higher plasma D-dimer levels (*P*<0.0001). Patients in group B had elevated levels in 7 out of 23 (30.5%) whereas in group C 75 out of 84 (89%) had increased D-dimer levels. Within group C, D-dimer levels were higher in patients with higher tumour load. The differences between these three groups were all highly significant (*P*<0.0001). Similarly D-dimer levels were higher in patients with rapid progressive disease compared to the slowly progressive group. Of the other variables number of sites (more than 2 *vs* 1; *P*=0.002) and the presence or absence of liver metastasis (*P*=0.032) were significantly related to higher D-dimer levels. Tumour type, differentiation, receptor status, menopausal status at diagnosis, prior treatment, were not significantly related to D-dimer level. Of all factors examined, only D-dimer levels showed a significant correlation with both number of sites involved and an estimation of load of disease in one of the three sites mentioned.

Fibrinogen levels were clearly higher in breast cancer patients, and the same factors that were correlated to higher D-dimer levels, were related to high fibrinogen levels. The plasma fibrinogen levels for the entire group of 107 breast cancer patients was 382±130 mg ml^−1^ (median 351 mg ml^−1^) (*P*=0.05). Similarly, within group C, high fibrinogen levels were related with tumour load (*P*=0.01), rapid progression (*P*=0.0007) and presence of liver metastasis (*P*=0.03). A relation between fibrinogen level with number of sites involved was not detected, which is clearly different from the results obtained with D-dimer levels. Furthermore, a strong statistical correlation exists between D-dimer levels and fibrinogen (*P*<0.0001).

Platelet counts were measured in all 107 patients with a mean value of 240±75×10^9^ per ml (median 243; range 65–616×10^9^ per ml). The number of platelets was not different between both groups of breast cancer patients. Within group C however, platelet counts were higher in the group with fast progressive disease compared to the group with slow progressive disease. Platelet count was not related to tumour type, differentiation, receptor status, menopausal status at diagnosis, age at diagnosis, disease-free interval, prior treatment, volume of disease, number of sites involved, nor presence or absence of liver metastasis.

### Serum IL-6, VEGF, VEGF per 10^6^ platelets and bFGF and breast cancer stage

Mean serum IL-6 levels for all 107 patients was 10.1±19.6 pg ml^−1^ (median 4.3; range 0.4–162.3 pg ml^−1^). These levels were significantly higher compared to the healthy control group. Serum IL-6 levels were also more elevated in the metastatic group compared to group B with a mean value of respectively 12.3±21.6 pg ml^−1^ and 2.0±1.1 pg ml^−1^ (*P*<0.0001). In the group of 58 patients with known progression kinetics estimates, 40 patients had IL-6 levels higher than 1.3 pg ml^−1^. Of these 40 patients, 32 were found in the fast progressive group and eight in the slow progressive group, whereas patients with IL-6 levels less than 1.3 (*n*=9), only four were found in the fast progressive group and five in the slow progressive group (χ^2^ test; *P*=0.033). Within group C patients, IL-6 levels were higher in the group of patients with liver metastasis, compared to those with (only) other metastasis (*P*=0.007). Serum IL-6 was also significantly higher in patients with more extensive disease (*P*=0.01).

The mean serum VEGF for both groups was 349±271 pg ml^−1^ (median 256 pg ml^−1^). In group C the mean serum VEGF was significantly higher than in group B patients. In group C, 47 out of 84 patients had increased serum VEGF levels, whereas only seven out of 23 of the patients with stage I or II breast cancer had increased serum VEGF levels (χ^2^ test; *P*=0.04). Within group C, patients with either high tumour load, more than two sites of metastatic deposits, or rapid progression tended to have higher serum levels, but these differences never reached clear statistical significance with respective *P* values of 0.08, 0.09 and 0.089. A significant linear relationship between serum VEGF and platelet count was observed in the entire group of breast cancer patients (Spearman correlation=0.33; *P*=0.0007). The calculated VEGF load (pg) per 10^6^ was significantly higher in the 107 breast cancer patients compared to our controls. But similarly as for serum VEGF, VEGF load per 10^6^ platelets was significantly higher in patients with progressive metastatic disease. Of patients with VEGF per 10^6^ platelets higher than 1, which is the 95th percentile for normal individuals (*n*=35), 29 were found in the fast progressive group, whereas patients with VEGF per 10^6^ platelets less than 1 (*n*=14), only seven were found in the fast progressive group (χ^2^ test; *P*=0.019). Serum bFGF had a mean level 10.11±42.31 pg ml^−1^ (median 4.00; range 0.50–351.0). The value of 10.11 pg ml^−1^ was however significantly higher than the one observed in our female control group (*P*=0.001). No differences were found however in the different groups of patients with breast cancer, or within the group of metastatic breast cancer patients.

### Relationship between markers of increased fibrinogen metabolism and angiogenic cytokines in patients with breast cancer

In both groups of breast cancer patients plasma levels of D-dimers are significantly elevated compared to healthy controls. Similarly the D-dimer levels are positively related to fibrinogen levels. Results of a more detailed correlation analysis of the different variables from the group of 107 breast cancer patients (group B+C) are given in Table 5

**Table 5:**
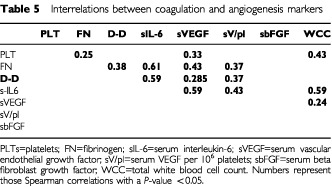
Interrelations between coagulation and angiogenesis markers

. In this group sIL-6 was most strongly correlated with plasma fibrinogen, plasma D-dimers, serum VEGF, and sVEGF per 10^6^ platelets. Between D-dimer levels and serum IL-6 (*P*<0.0001) and similarly between plasma fibrinogen and serum IL-6 (*P*<0.0001) a highly significant positive correlation is observed. A statistically significant correlation was found between serum VEGF and IL-6 (*P*<0.0001) and between the calculated VEGF load per 10^6^ platelets and IL-6 (*P*<0.0001).

D-dimer levels show a strong correlation with serum VEGF (*P*=0.0016), and VEGF load per 10^6^ platelets (*P*<0.0001).

### Prognostic studies

**Figure 1:**
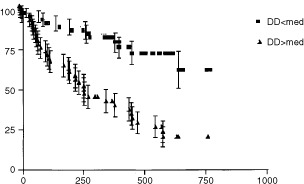
Overall survival (days) in 84 patients with metastatic breast cancer according to D-dimer levels.

In univariate analysis in overall survival (OS) studies, DD level was a prognostic parameter with high concentrations being associated with worse survival (Figure 1

). Tumour differentiation, hormone receptor status, stage at presentation, number of sites involved, presence of visceral disease, serum IL-6, plasma VEGF, serum fibrinogen were also prognostic parameters. For Cox multivariate analysis in OS studies, only DD, fibrinogen and presence of visceral disease remained significant.

## DISCUSSION

Tumour expansion depends on the proliferation characteristics of the tumour cells and the interaction with different stromal cells and supportive tissue. This stromal remodelling utilizes two intrinsically related physiological mechanisms, both firmly inhibited under resting conditions: the system of haemostasis and fibrinolysis and the process of angiogenesis. Tissue markers of fibrinolysis, mainly the levels of tissue-type (tPA) and urokinase-type (uPA) plasminogen activators, have prognostic significance in patients with localized and metastatic breast cancer (Solomayer *et al*, 1997). In patients with both non-small cell and small cell lung cancer, increased plasma D-dimer levels were shown to be predictive for a worse clinical outcome (Taguchi *et al*, 1997). Similarly in patients with primary colorectal cancer, D-dimers predicted for more advanced stage of disease and worse prognosis (Oya *et al*, 1998). Plasma D-dimer levels predict for the presence of lymph node involvement in patients with operable breast cancer (Blackwell *et al*, 2000). This analysis of D-dimer levels in breast cancer patients is taken a step further in this study by investigating their significance in patients with documented metastatic and progressive disease. The D-dimers were found to be increased in 75 of 84 (89%) of these patients. The mean level of D-dimers in this population was in the range of 2.5 μg ml^−1^. Increased levels were not shown to be related with histological subtype, hormone receptor status, disease-free interval, number and type of prior treatment for metastatic disease, or patient age. However, load of disease (*P*<0.0001), clinical progression rate (*P*<0.0001), number of involved sites (*P*=0.04), and presence of liver metastasis (*P*=0.032) were related to increased D-dimers. Furthermore increased D-dimers were predictive of a worse prognosis both in univariate and multivariate analysis. Fibrinogen levels were significantly increased and showed a strong positive correlation with D-dimers. This suggests that in unselected breast cancer patients, fibrinogen is actively being converted into fibrin and that fibrinolysis results in increased D-dimer levels. As these fibrin degradation products are related to the clinically measured growth rate, they might be in part a reflection of ongoing fibrinogen metabolism within the actively remodelled tumour stroma. Because the amount of these degradation products is the only parameter to be strongly related to both tumour bulk and the number of involved sites, it seemed justified to consider D-dimer levels in this setting as a marker for the disease load.

In breast cancer patients serum IL-6 has been shown to correlate with increasing numbers of involved sites, presence of liver metastasis, and disease progression. Serum IL-6 was an independent prognostic factor in a multi-variate analysis in patients with metastatic disease (Zhang and Adachi, 1999). In 60 of the 84 (72%) patients with metastatic disease serum IL-6 was increased. As in the series by Zhang and Adachi (1999) serum levels of IL-6 were higher in patients with liver metastases. Because IL-6 has both a thrombopoietic and a VEGF upregulating activity, it was postulated that serum VEGF might be influenced by IL-6 (Cohen *et al*, 1996; Möhle *et al*, 1997). A positive correlation was shown to exist between serum IL-6 levels and serum VEGF and the calculated load of VEGF per 10^6^ platelets (Salgado *et al*, 2000). These observations are confirmed in this group of patients, both between IL-6 and serum VEGF and between IL-6 and the calculated load of VEGF per 10^6^ platelets, corroborating the potential role of IL-6 on the expression of VEGF in platelets. This study now extends the interrelationships of serum IL-6 with fibrinogen and D-dimer levels. On the other hand, serum IL-6 is less influenced by the number of organs involved, but is influenced by both volume of disease, and even more so it is a critical predictor of progression kinetics (*P*=0.01) which is in accordance with earlier studies (Zhang and Adachi, 1999). Serum IL-6 seems to influence both sVEGF and sVEGF per platelets, as well as plasma fibrinogen and D-dimer levels. Serum IL-6 levels are not different between the slow and rapidly growing group, but the distribution of patients with high and low serum IL-6 levels was significantly related with progression kinetics. IL-6 might be a critical component of VEGF expression locally at the site of a growing tumour, but furthermore increasing the amount VEGF stored in platelets. The earlier observations on the relationship between serum VEGF and platelet count are confirmed. Patients with high calculated VEGF per pl, above the 95th percentile of healthy adults, tend to be in the fast progressive group, whereas patients with low VEGF per pl tend to be in the slowly progressive group. These data suggest that platelets might contribute to angiogenesis, by transporting increased amounts of VEGF to sites selected by a pro-coagulant environment, c.q. areas of active angiogenesis similarly to the contribution of platelets in wound healing (Knighton *et al*, 1982).

If one assumes that active coagulation also occurs extravascularly and contributes to stromal remodelling, then the observed correlations between the cytokine levels, fibrin split products and tumour kinetics, suggest that as new microvessels develop, they are rendered more permeable for large molecules by VEGF, either locally produced or delivered by platelets, enabling a shift towards the extravascular compartment of plasma molecules like plasminogen and fibrinogen. Extravasated fibrinogen is then converted by the tissue-factor activated extrinsic coagulation pathway to cross-linked fibrin. The presence of active plasmin in the tumour stroma cleaves fibrin into a number of degradation products.

In conclusion this study extends the significance of increased plasma D-dimers as a predictor for more rapid tumour growth, the presence of more widespread disease and for shorter survival in breast cancer patients. It also suggests an interaction between the coagulation system and the mediators of angiogenesis and the role of interleukin-6 deserves further investigations.
